# Integrative Studies on a New Ciliate *Campanella sinica* n. sp. (Protista, Ciliophora, Peritrichia) Based on the Morphological and Molecular Data, With Notes on the Phylogeny and Systematics of the Family Epistylididae

**DOI:** 10.3389/fmicb.2021.718757

**Published:** 2021-07-30

**Authors:** Zhe Wang, Tong Wu, Borong Lu, Yong Chi, Xue Zhang, Saleh A. Al-Farraj, Weibo Song, Alan Warren, Lifang Li, Chundi Wang

**Affiliations:** ^1^Marine College, Shandong University, Weihai, China; ^2^Key Laboratory of Mariculture, Ministry of Education, Institute of Evolution and Marine Biodiversity, Ocean University of China, Qingdao, China; ^3^Department of Zoology, College of Science, King Saud University, Riyadh, Saudi Arabia; ^4^Laboratory for Marine Biology and Biotechnology, Qingdao National Laboratory for Marine Science and Technology, Qingdao, China; ^5^Department of Life Sciences, Natural History Museum, London, United Kingdom

**Keywords:** rDNA sequences, morphology, peritrichs, sessilid ciliate, phylogenetic analyses

## Abstract

During an investigation on freshwater peritrichs, a new colonial sessilid ciliate, *Campanella sinica* n. sp., was isolated from aquatic plants in an artificial freshwater pond in Qingdao, China. Specimen observations of this species were performed both *in vivo* and using silver staining. *C. sinica* n. sp. is characterized by the appearance of the mature colony, which is up to 2 cm high and contains more than 1,000 zooids, the asymmetric horn-shaped zooids, strongly everted and multi-layered peristomial lip, the slightly convex peristomial disc, and the well-developed haplokinety and polykinety, which make more than four circuits of the peristome before descending into the infundibulum. The small subunit ribosomal DNA (SSU rDNA), 5.8s rDNA and its flank internal transcribed spacers (ITS1-5.8s rDNA-ITS2), and large subunit ribosomal DNA (LSU rDNA) are sequenced and used for phylogenetic analyses which reveal that the family Epistylididae Kahl, 1933 is non-monophyletic whereas the genus *Campanella* is monophyletic and nests within the basal clade of the sessilids. The integrative results support the assertion that the genus *Campanella* represents a separate lineage from other epistylidids, suggesting a further revision of the family Epistylididae is needed. We revise *Campanella* including the transfer into this genus of a taxon formerly assigned to *Epistylis*, which we raise to species rank, i.e., *Campanella ovata* (Nenninger, 1948) n. grad. & n. comb. (original combination *Epistylis purneri* f. *ovata* Nenninger, 1948). In addition, we provide a key to the identification of the species of *Campanella*.

## Introduction

Ciliated protists (ciliates) have a widespread distribution and are found almost everywhere on the earth’s surface where there is sufficient water for their survival (Lynn, 2008; Zhao et al., 2020; Huang et al., 2021; Liu et al., 2021). Members of the subclass Peritrichia Stein, 1859 are characterized by their well-developed oral ciliature and vestigial somatic ciliature and are divided into two orders, Sessilida Kahl, 1933 and Mobilida Kahl, 1933 (Song et al., 2009; Gao et al., 2016; Wang et al., 2017a, 2018, 2019, 2020; Hu et al., 2019; Lu et al., 2019; Chen et al., 2020b; Wu et al., 2020, 2021). Sessilids are widely distributed in a variety of aquatic environments where they play an important role as consumers of bacteria and other microorganisms (Lynn, 2008; Shen and Gu, 2016). At least 800 species of sessilids, representing about 110 genera, have been described, although Foissner et al. (2010) estimated the number of genera to be 105–140 (Lynn, 2008; Song et al., 2009; Lu et al., 2019, 2020; Wu et al., 2020). Knowledge and understanding of the diversity, taxonomy, systematics, and evolution of sessilids are rapidly advancing with the increased application of methods, such as differential interference contrast microscopy, silver staining, and molecular phylogenetic analysis (Gentekaki et al., 2017; Lu et al., 2019, 2020; Wu et al., 2020). However, only a small proportion of sessilid species have been investigated using such methods (Zhuang et al., 2018; Jiang et al., 2019; Wu et al., 2020).

Recent molecular phylogenetic analyses have cast doubts on the validity, monophyly, and/or membership for several peritrich genera and families, for example, the family Epistylididae Kahl, 1933, species of which are distributed among several separate clades in gene trees (Miao et al., 2001, 2004; Sun et al., 2016; Zhuang et al., 2018). According to Lynn (2008), the family Epistylididae comprises 11 genera and is mainly characterized by the sessile trophont that: (1) is attached to substrate *via* either a non-contractile stalk or the scopula; (2) has an everted peristomial lip and a slightly projecting peristomial disc; and (3) has an oral ciliature that makes more than one turn around the peristome before entering the infundibulum (Lynn, 2008; Irwin and Lynn, 2015; Lu et al., 2020). One of the most distinctive genera of epistylidids is *Campanella* Goldfuss, 1820. *Campanella* is colonial with a non-contractile stalk and large zooids that have a multi-layered peristomial lip, an oral ciliature that makes several circuits around the peristome before descending into the infundibulum and a reticulate silverline system (Nenninger, 1948; Yu et al., 1995; Lynn, 2008). Three valid species of *Campanella* have been described, namely *Campanella umbellaria* (Linnaeus, 1758) Goldfuss, 1820 (the type species), *C. hanchuansensis* Yu et al., 1995 and *C. purneri* (Nenninger, 1948) Yu et al., 1995, but only *C. umbellaria* has been studied using modern methods (Miao et al., 2004; Shi et al., 2004; Wang et al., 2011). Nevertheless, phylogenetic analyses based on small subunit ribosomal DNA (SSU rDNA) sequences have challenged the traditional classification of *Campanella* suggesting that this genus may not belong to the family Epistylididae (Miao et al., 2004; Utz et al., 2010; Sun et al., 2016).

In the present study, we describe a new species, *C. sinica* n. sp., isolated from a freshwater pond in Qingdao, China, based on observations of specimens both *in vivo* and following silver staining. The phylogeny of this species based on SSU rDNA, ITS1-5.8S rDNA-ITS2, and large subunit ribosomal DNA (LSU rDNA) sequences is also analyzed, offering new insights into the evolutionary relationships of sessilids. Moreover, we reevaluate the classification of epistylidid-like species based on the morphological and phylogenetic results. In addition, the taxonomy and nomenclature of the species of *Campanella* are reviewed and a key to their identification is supplied.

## Materials and Methods

### Sampling and Observation

**Figure 1:**
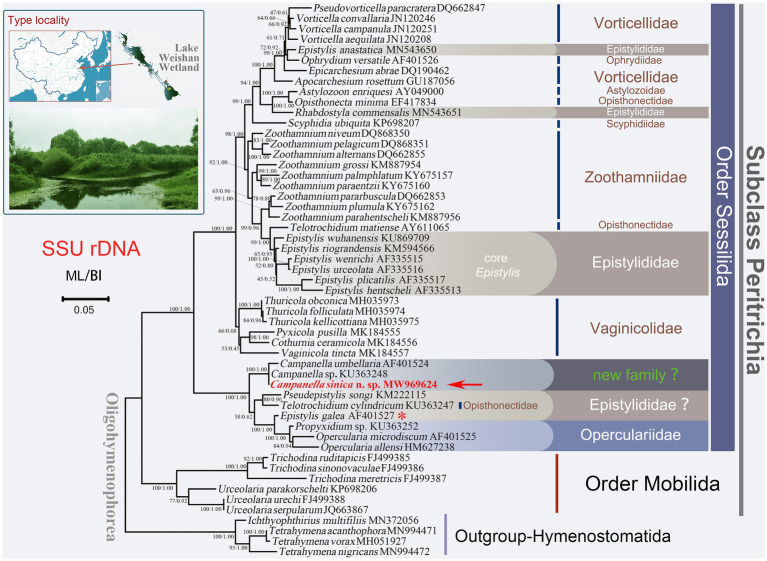
Maximum likelihood (ML) tree inferred from small subunit ribosomal DNA (SSU rDNA) sequence data. Sequence of *Campanella sinica* n. sp. is in red and indicated by an arrow. Numbers given at nodes of branches are the ultrafast bootstrap percent (UF) values for ML analysis and posterior probability (PP) values for BI analysis. The scale bar corresponds to five substitutions per 100 nucleotide positions. The asterisk represents the validity of this sequence needs to be verified.

*Campanella sinica* n. sp. was collected between 27 March and 27 July 2020 from aquatic plants growing in an artificial freshwater pond (N36°03ꞌ45ꞌꞌ; E120°20ꞌ10ꞌꞌ) in Ocean University of China, Qingdao, China (Figure 1, inset). The water and sediment in the pond came from the Lake Weishan wetland (N34°43ꞌ59ꞌꞌ; E117°9ꞌ22ꞌꞌ). Live cells were observed and measured under a Zeiss AXIO Imager D2 microscope (Zeiss, Germany). The ciliature and silverline system were revealed by the protargol staining method and dry silver nitrate staining method, respectively (Klein, 1958; Foissner, 2014). The protargol powder was synthesized following the method of Pan et al. (2013). All measurements were performed at 400–1,000× magnifications. Drawings of live cells, ciliature, and silverline system were based on direct observations and photomicrographs. Classification and terminology are mainly according to Warren (1986) and Lynn (2008), respectively.

### DNA Extraction, PCR Amplification, and Sequencing

One to five zooids were isolated and washed several times in double distilled water using glass micropipettes under a dissecting microscope to remove contamination. Genomic DNA was extracted following the methods described by Zhang et al. (2020). The SSU rDNA, ITS1-5.8S rDNA-ITS2, and partial LSU rDNA sequences were amplified using primers 82F (5ꞌ-GAA ACT GCG AAT GGC TC-3ꞌ), 18SR (5ꞌ-TGA TCC TTC TGC AGG TTC ACC TAC-3ꞌ), 5.8SF (5ꞌ-GTA GGT GAA CCT GCG GAA GGA TC-3ꞌ), and R3 (5ꞌ- CAT TCG GCA GGT GAG TTG TTA CAC -3ꞌ), respectively (Medlin et al., 1988; Gong et al., 2007; Huang et al., 2018). Q5® Hot Start High-Fidelity DNA Polymerase (New England BioLabs, United States) was used for PCR to minimize the possibility of amplification errors. The fragments were sequenced biodirectionally by the Tsingke Biological Technology Company (Qingdao, China).

### Phylogenetic Analyses

**Figure 2:**
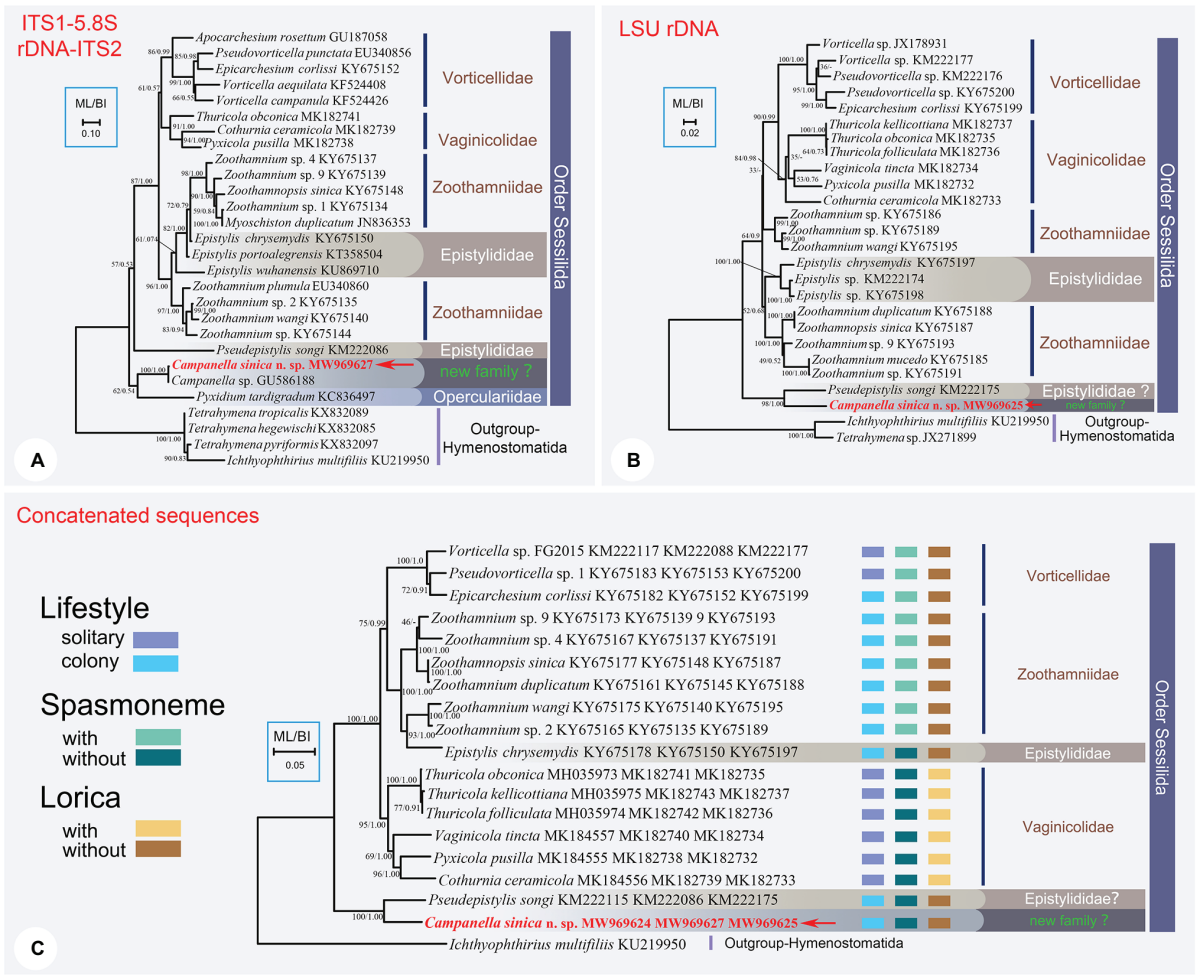
The ML trees inferred by ITS1-5.8S rDNA-ITS2 region **(A)**, LSU rDNA sequence **(B)**, and the concatenated data of SSU rDNA, ITS1-5.8S rDNA-ITS2, and LSU rDNA sequences **(C)**. In each tree, *Campanella sinica* n. sp. is in red and indicated by an arrow. Numbers given at nodes of branches are the UF values for ML analysis and PP values for BI analysis. The scale bar corresponds to ten, two, and five substitutions per 100 nucleotide positions in **(A-C)**, respectively.

Phylogenetic analyses were performed both with single-gene datasets of SSU rDNA, ITS1-5.8S rDNA-ITS2, and LSU rDNA separately, and with a concatenated dataset of all three genes. In addition to the newly obtained sequences, other sequences downloaded from the GenBank database (for accession numbers, see Figures 1, 2), selected based on earlier studies (Sun et al., 2016; Zhuang et al., 2018), were used in the phylogenetic analyses. The boundary of the ITS1-5.8S rDNA-ITS2 was identified according to the methods described by Sun et al. (2010, 2013). A total of 53 SSU rDNA sequences including *Campanella sinica* n. sp., 48 peritrichs and four hymenostomatids as outgroup taxa were used to construct the SSU rDNA trees, while the analyses of ITS1-5.8S rDNA-ITS2, LSU rDNA, and concatenated data contained 28, 26, and 19 taxa, respectively (Figures 1, 2). Before constructing the trees, the selected sequences were aligned by MAFFT and further refined by Guidance 2 (https://guidance.tau.ac.il/ver2/; Katoh and Standley, 2013; Sela et al., 2015). The final alignments used for subsequent phylogenetic analyses comprised 1,605 sites for the SSU rDNA, 521 sites for the ITS1-5.8S rDNA-ITS2, 1,749 sites for the LSU rDNA, and 3,702 sites for the concatenated (SSU rDNA-ITS1-5.8S rDNA-ITS2-LSU rDNA) dataset.

The best-fit models for maximum likelihood (ML) and Bayesian inference (BI) analyses were calculated by ModelFinder under Bayesian Information Criterion (Table 1; Kalyaanamoorthy et al., 2017). The concatenated sequences were treated with partitioned analyses to give the best-fit models for different regions.

**Table 1:** The best-fit substitution model selected for different phylogenetic dataset.

	Region	IQ-TREE	MrBayes
Single gene tree	SSU rDNA	TIM2 + F + R3	GTR + I + G
	ITS1-58S rDNA-ITS2	TIM2 + F + I + G4	GTR + I + G
	LSU rDNA	TIM2 + F + I + G4	GTR + I + G
Concatenated gene tree	SSU rDNA	TN + F + R2	HKY + G
	ITS1-58S rDNA-ITS2	HKY + F + G4	GTR + I + G
	LSU rDNA	TIM2 + F + I + G4	GTR + I + G

Maximum likelihood analysis was carried out by IQ-TREE v2.0 with 10000 Ultrafast bootstrap replicates (Minh et al., 2020). BI analysis was carried out by MrBayes v3.2.7 (Ronquist et al., 2012). Markov chain Monte Carlo simulations were run for 1,000,000 generations with sampling every 100 generations. The first 25% of trees were discarded as burn-in. The run continued until the SD of split frequencies was below 0.01, and the effective sample size was > 200 (Rambaut et al., 2014). Phylogenetic trees were viewed by MEGA v.7 (Kumar et al., 2016) or Figtree.^1^

### Sequence Comparisons Between *Campanella sinica* n. sp. and Its Closely Related Congeners

**Figure 3:**
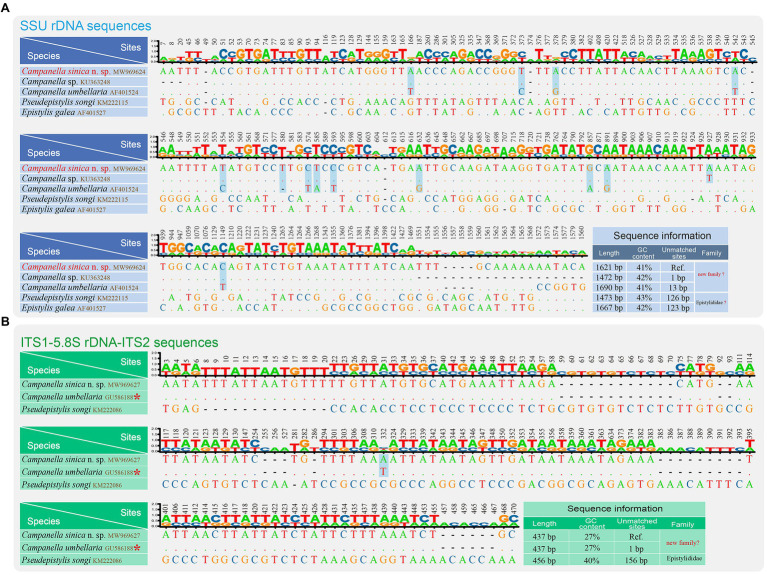
Nucleotide differences among *Campanella sinica* n. sp. and closely related taxa based on SSU rDNA sequences **(A)** and ITS1-5.8S rDNA-ITS2 sequences **(B)**. The numbers in the header indicate the unmatched site positions. The sequences of *Campanella sinica* n. sp. in this study are shown in red. The variable sites of *C. sinica* n. sp. and the other *Campanella* species are shown in gray. Ref., reference sequence. The asterisk indicates the validity of this sequence needs to be verified.

The SSU rDNA and ITS1-5.8S rDNA-ITS2 sequences of *C. sinica* n. sp. were separately aligned with closely related taxa by MAFFT v.7 with L-INS-I strategy (Katoh and Standley, 2013). The ends of alignments were refined by eye. The final comparisons were edited and viewed by Bioedit v.7 (Hall, 1999) and TBtools (Chen et al., 2020a). The nucleotide differences between each of the new sequences and those species with high sequence similarities are shown in Figure 3.

## Results

### ZooBank Registration

Present work: urn:lsid:zoobank.org:pub:7D581595-D090-4750-B26B-27B1033356FF.

*Campanella sinica* n. sp.: urn:lsid:zoobank.org:act:D25C2BDA-EB29-457D-8DC0-B7CF4C62B72.
Subclass Peritrichia Stein, 1859
Order Sessilida Kahl, 1933
Family Epistylididae Kahl, 1933
Genus *Campanella* Goldfuss, 1820
*Campanella sinica* n. sp.

#### Diagnosis

Mature colony up to 2 cm high with up to 1,000 zooids and dichotomously branched stalk. Zooids asymmetric horn-shaped, about 125–240 × 80–120 μm *in vivo*. Peristomial lip strongly everted. Haplokinety and polykinety makes 4.5–5 circuits around peristome before descending into infundibulum. Peristomial disc slightly convex. Contractile vacuole ventrally located beneath peristomial lip. Macronucleus C-shaped, transversely oriented. Infundibular polykineties 1 and 2 (P1, P2) each with three equal-length rows, infundibular kinety 3 (P3) with up to six rows. Abstomal end of P3 converges with P2. Transverse silverlines numbering about 42–48 from peristome to trochal band and about 26–28 from trochal band to scopula.

#### Type Locality and Ecological Features

Lake Weishan wetland (N34°43ꞌ59ꞌꞌ; E117°9ꞌ22ꞌꞌ), a freshwater wetland in Jining, Shandong Province, China. Water temperature of the artificial pond: 20°C.

#### Deposition of Slides

**Figure 4:**
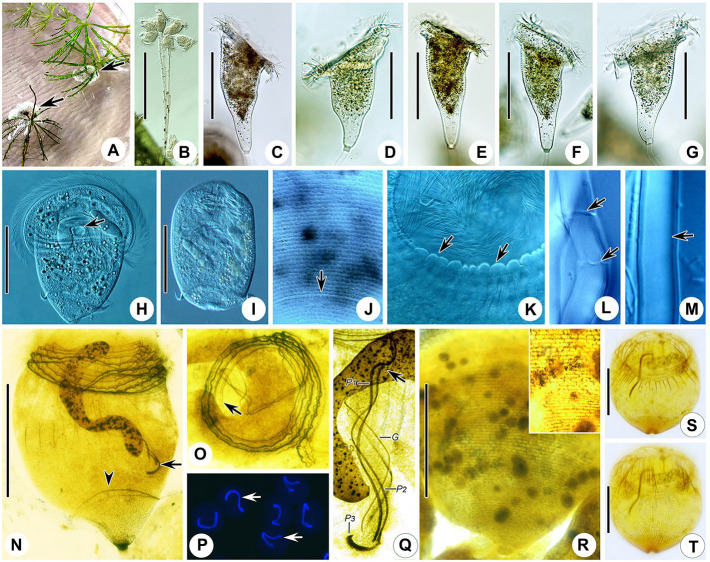
Photomicrographs of *Campanella sinica* n. sp. *in vivo* **(A–M)**, after protargol staining **(N,O,Q,S,T)**, after Hoechst-33342 staining **(P)**, and after dry nitrate silver staining **(R)**. **(A)** Colony (arrows) on aquatic plant. **(B)** Immature colony. **(C–G)** Different individuals, showing the variation of zooid shape. **(H)** A compressed zooid, arrow indicates the contractile vacuole. **(I)** Telotroch. **(J)** Pellicular striations, arrow indicates the aboral trochal band. **(K)** Detail of peristomial region of a contracted zooid, arrows indicate the peristomial lip. **(L,M)** Showing that the stalk is hollow (arrows). **(N)** Holotype specimen, showing the infraciliature and macronucleus, arrow indicates P3, arrowhead indicates the aboral trochal band. **(O)** Oral ciliature, apical view, arrow indicates the beginning of the oral ciliature. **(P)** Showing the macronucleus (arrows). **(Q)** Infundibular polykineties, arrow marks the anterior end of P2. **(R)** Silverline system. **(S,T)** Myoneme system. G, germinal kinety; P1–3, infundibular polykineties 1–3. Scale bars = 80 μm.

One protargol slide with the holotype specimen circled in ink (registration number: WT2020072201-01; Figure 4N), and two “dry” silver nitrate slides with paratype specimens (registration numbers: WZ2020052701-01, 02) were deposited in the Laboratory of Protozoology, Ocean University of China, Qingdao, China.

#### Etymology

The species-group name “*sinica*” refers to the country where the sample was collected.

### Description

**Figure 5:**
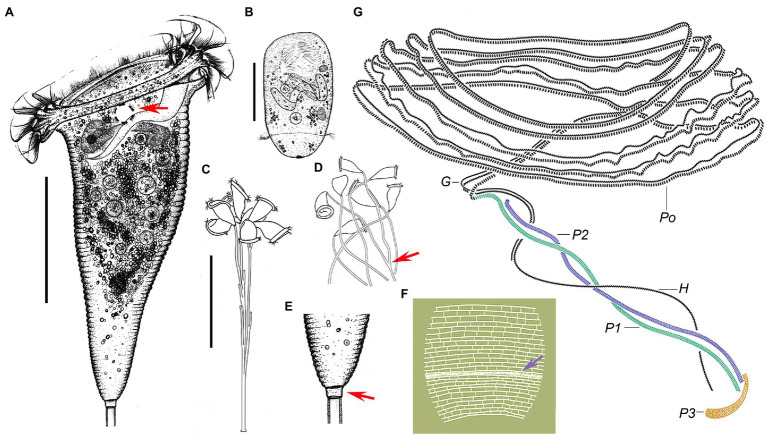
*Campanella sincia* n. sp. *in vivo* **(A–E)**, after dry silver nitrate staining **(F)** and protargol **(G)** staining. **(A)** Typical zooid, arrow indicates the contractile vacuole. **(B)** Teletroch. **(C,D)** Immature colonies at low magnification, showing the branching pattern of the stalk, arrow in **(D)** shows an example of dichotomous branching. **(E)** Junction of stalk and zooid at high magnification, arrow shows the scopula. **(F)** Partial silverline system, arrow marks trochal band. **(G)** Oral ciliature. G, germinal kinety; H, haplokinety; Po, polykinety; and P1–3, infundibular polykineties 1–3. Scale bars: 50 μm in **(A,B)**; 100 μm in **(C)**.

Zooids about 125–240 × 80–120 μm *in vivo*, asymmetrical horn-shaped, gradually tapering to scopula (Figures 4B–G, 5A; Table 2). Peristomial lip multi-layered, about 125–145 μm in diameter *in vivo*, relatively thick and strongly everted (Figures 4C–G, 5A). Peristomial disc slightly convex and elevated above peristome in fully extended zooids (Figures 4C–G, 5A). Oral cilia sturdy, about 12 μm long (Figures 4C–H, 5A). Telotroch cylindrical, with peristomial region fully contracted (Figures 4I, 5B).

**Table 2:** Morphometric data of *Campanella sinica* n. sp.

Character	Min	Max	Mean	*SD*	CV	*N*
Zooid length, *in vivo*^a^	125	240	184	36.2	19.7	10
Zooid width, *in vivo*^a^	80	120	98	13.8	14.1	10
Diameter of peristomial lip, *in vivo*^a^	125	145	131.5	6.7	5.1	10
Number of silverlines, peristome to aboral trochal band^b^	42	48	45.7	2.4	5.3	7
Number of silverlines, aboral trochal band to scopula^b^	26	28	27.7	0.8	2.7	7

a All measurements are in μm.

b Rough data.

Min, minimum; Max, maximum; *SD*, standard deviation; CV, coefficient of variation in %; Mean, arithmetic mean; and *N*, number of specimens.

Cytoplasm colorless, usually contains several grayish/darkish granules, 7 μm in diameter (Figures 4C, 5A). Contractile vacuole about 15 μm in diameter, located at ventral wall of infundibulum beneath peristomial lip (Figures 4H, 5A). Macronucleus relatively large, irregular C-shaped, transversely oriented in anterior half of zooid (Figures 4H,N,P, 5A). Pellicular striations reticulate, conspicuous when viewed at magnifications above 200× (Figures 4J,K,R, 5F). Trochal band dikinetid and located three-quarters of the way down length of zooid (Figures 4J, 5F). Scopula prominent, same width as stalk (Figures 4C–G, 5A,E).

Mature colony up to 2 cm high, contains more than 1,000 zooids (Figure 4A). Stalk straight, smooth, and hollow (Figures 4L,M, 5A,E), dichotomously branched with zooids regularly located in pairs (Figures 4B, 5C,D). Colony-founding zooid often sits on a relatively short stalk (Figure 5D).

Haplokinety and polykinety make approximately 4.5–5 circuits around peristome before entering infundibulum where they make a further circuit (Figures 4N,O, 5G). Polykinety transforms into infundibular polykinety 1 (P1). P1 accompanied by infundibular polykineties 2 and 3 (P2 and P3) within infundibulum. P1 and P2 composed of three equal-length rows of kinetosomes (Figures 4Q, 5G). P3 composed of about six rows (rough data) of kinetosomes (Figures 4Q, 5G). Germinal kinety (G) lies parallel to haplokinety (H) in abstomal region of infundibulum. Filamentous reticulum (FR) lies beneath wall of infundibulum parallel with haplokinety. Adstomally, P3 extends far beyond ends of P1 and P2, and sharply curves to make nearly 1/2 circuit around margin of cytostome (Figures 4Q, 5G).

Silverline system reticulate, with 42–48 (*n* = 7) transverse silverlines from peristome to aboral trochal band, and 26–28 (*n* = 7) transverse silverlines from aboral trochal band to scopula (Figures 4R, 5F). Transverse silverlines in anterior region of cell slightly more widely spaced than those in posterior region (Figures 4R, 5F). Myoneme system complex, composed of longitudinal fibers, medium-length longitudinal fibers, short longitudinal fibers, linking fibers, and support fibers (Figures 4S,T).

### Phylogenetic Analyses

The GenBank accsssion numbers, lengths, and GC contents of the newly obtained rDNA sequences (SSU rDNA, ITS1-5.8S rDNA-ITS2, and LSU) are shown in Table 3.

**Table 3:** Sequence information of *Campanella sinica* n. sp.

	GenBank Acc. No.	Length	GC content
SSU rDNA	MW969624	1,621	41%
ITS1-58S rDNA-ITS2	MW969627	438	27%
LSU rDNA	MW969625	1,148	40%

In all the cases, the ML and BI trees have similar topologies, therefore, only the ML tree is presented for each gene or region. In the SSU rDNA tree, the order Sessilida is monophyletic, whereas the families Epistylididae, Opisthonectidae Foissner, 1976, Vorticellidae Ehrenberg, 1838, and Zoothamniidae Sommer, 1951 were non-monophyletic (Figure 1). *Campanella* species (*C. umbellaria*, *C. sinica* n. sp., and *Campanella* sp.) formed a fully supported clade (ML/BI, 100/1.00) that was sister to a clade comprising two epistylidids (*Epistylis galea* Ehrenberg, 1831 and *Pseudepistylis songi* Peng et al., 2007), three opercularids (*Opercularia microdiscum* Fauré-Fremiet, 1904, *O. allensi* Stokes, 1887, and *Propyxidium* sp.), and the opisthonectid *Telotrochidium cylindricum* (Figure 1). The above-mentioned species formed the basal clade within the Sessilida with maximal support (ML/BI, 100/1.00). The remaining sessilids formed a clade with maximal support (ML/BI, 100/1.00). Within this clade, loricate species of the family Vaginicolidae formed the basal branch, albeit with low support (ML/BI, 66/0.68). Species of the family Zoothamniidae were divided into separate clades. The majority of epistylidids (core *Epistylis* species) nested within Zoothamniidae (Figure 1), while *E. anastatica* (Linnaeus, 1767) Ehrenberg, 1830 and another epistylidid (*Rhabostyla commensalis* Möbius, 1888) nested within the Vorticellidae, and *E. galea* Ehrenberg, 1831 grouped with the operculariids, although the identity of this latter sequence awaits verification (Figure 1).

The topology of the ITS1–5.8S rDNA–ITS2 tree was broadly similar to that of the SSU rDNA tree except the position of family Vaginicolidae, which clustered with the Vorticellidae to form the crown group of sessilids rather than branching basally. Three colonial species, *Campanella*, *C. sinica* n. sp., and *Propyxidium taradigradum* Van der Land, 1964, formed the basal clade of the Sessilida. The remaining sessilids clustered into similar groups as those in the SSU rDNA tree. Each of the families Zoothamniidae, Epistylididae, Operculariidae, and Vorticellidae was non-monophyletic (Figure 2A).

In the LSU rDNA tree, *Campanella sinica* n. sp. and *P. songi* formed a strongly supported clade (ML/BI, 98/1.00) which was basal within the Sessilida (Figure 2B). The topology of the concatenated sequence tree was similar to that of the SSU rDNA tree, supporting the assertion that *Campanella* and *Epistylis* are distantly related. *Epistylis chrysemydis* Bishop and Jahn, 1941 nested within the Zoothamnidae (Figure 2C).

We also compared nucleotide differences of SSU rDNA and ITS1-5.8S rDNA-ITS2 sequences between *C. sinica* n. sp. and other closely related taxa (Figure 3). The SSU rDNA sequence most similar to *C. sinica* n. sp. was *Campanella* sp. (KU363248) with one variable site, followed by *C. umbellaria* (AF01524) with 13 variable sites. The GC contents of the SSU rDNA sequences of *Campanella* species, *Epistylis gelea*, and *P. songi* ranged from 41 to 43% (Figure 3A).

The ITS1-5.8S rDNA-ITS2 sequence most similar to *C. sinica* n. sp. was *C. umbellaria* (GU586188) with one variable site, followed by *Pseudepistylis songi* (KM222086) with 156 variable sites. The GC contents of the ITS1-5.8S rDNA-ITS2 sequences of *Campanella* species and *P. songi* were 27 and 40%, respectively (Figure 3B).

## Discussion

### Review of *Campanella* Species

**Figure 6:**
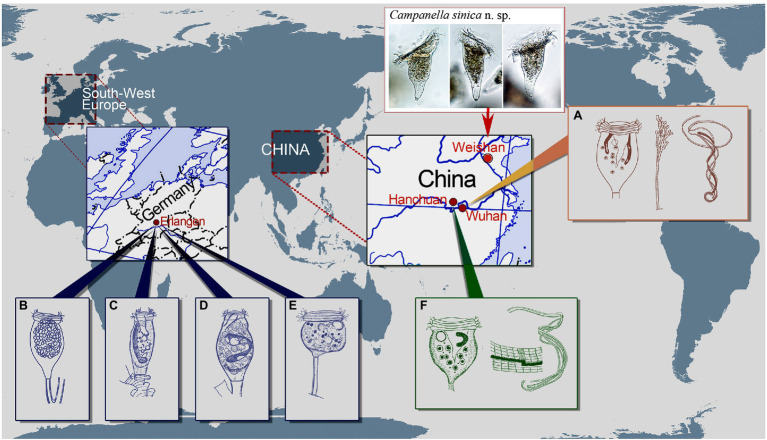
Schematic drawings of *Campanella* species showing some localities of *C. umbellaria* **(A,B,E)** and type localities of *C. purneri* **(C)**, *C. ovata* n. grad. & n. comb. **(D)**, and *C. hanchuanensis* **(F)**. **(A)** After Shi et al. (2004) and Shen and Gu (2016). **(B–E)** After Nenninger (1948). **(F)** After Yu et al. (1995) and Shen and Gu (2016).

*Campanella umbellaria* (Linnaeus, 1758) Goldfuss, 1820 is commonly found in eutrophic freshwaters (Linnaeus, 1758; Foissner et al., 1992). It is characterized by its symmetrical inverted bell-shaped zooid and horseshoe-shaped macronucleus (Foissner et al., 1992; Figures 6A,B). According to Sládeček and Sládečková (1974) and Foissner et al. (1992), the synonyms of this species are as follows: *Hydra umbellaria* Linnaeus, 1758, *Epistylis flavicans* Ehrenberg, 1838, *Epistylis grandis* Ehrenberg, 1838, *Epistylis tincta* Stokes, 1887, and *Epistylis liebmanni* Nenninger, 1948. It is noteworthy that no illustration of *E. tincta* was provided in the original description (Stokes, 1887). Kahl (1935) considered that *E. tincta* should belong to the genus *Campanella* and mentioned the difficulty in separating *E. tincta* from *C. umbellaria*. Yu et al. (1995) also accepted that *E. tincta* and *E. liebmanni* should belong to the genus *Campanella* but either overlooked or rejected the assertion that they are junior synonyms of *C. umbellaria*, referring to them as *C. tincta* and *C. liebermanni* (which is presumably a misspelling of “*liebmanni*”), respectively. Based on the descriptions provided by Stokes (1887) and Nenninger (1948), we agree with Sládeček and Sládečková (1974) and Foissner et al. (1992), who suggested that *E. tincta* and *E. liebmanni* (and as a consequence *C. tincta* and *C. liebermanni*) are junior synonyms of *C. umbellaria*. Nevertheless, variations are recognized in different populations of *C. umbellaria*, so it is possible that cryptic species might exist among these populations (Sládeček and Sládečková, 1974; Foissner et al., 1992). Further investigations including both morphological and molecular methods therefore need to be carried out to accurately characterize such populations.

Besides *E. liebmanni*, Nenninger (1948) described other two taxa that she assigned to the genus *Epistylis*, i.e., *E. purneri* and *E. purneri* f. *ovata*, both of which should belong to the genus *Campanella* due to the presence in each of a multi-layered peristomial lip and an oral ciliature that makes more than four circuits around peristome before descending into the infundibulum (Figures 6B–D). *Epistylis purneri* and *E. purneri* f. *ovata* are similar but they differ from each other as follows: (1) the zooid shape of *E. purneri* is cylindrical, whereas that of *E. purneri* f. *ovata* is pyriform and (2) the stalk of *E. purneri* has several conspicuous transverse annular bulges, whereas that of *E. purneri* f. *ovata* has smooth surface (Figures 6C,D). Consequently, *E. purneri* f. *ovata* should be elevated to species rank. Yu et al. (1995) established the name *C. purneri* for *E. purneri* although without providing any justification. Here, we establish *Campanella ovata* (Nenninger, 1948) n. grad. & n. comb for *E. purneri* f. *ovata* (original combination *Epistylis purneri* f. *ovata* Nenninger, 1948). Nenninger (1948) also reported a new sessilid species, *Opisthostyla tritora*, which resembles colony-founding individuals of *C. umbellaria* (Figure 6E). *Opisthostyla* Stokes, 1886 is characterized by its solitary lifestyle and long, non-contractile stalk that is curved near the point of attachment to its substrate and acts in a spring-like manner throwing the organism backward when the zooid contracts (Stokes, 1886; Shen and Gu, 2016). However, the stalk of *O. tritora* has neither the structure nor the behavior that characterizes *Opisthostyla*. Therefore, *O. tritora* was probably misidentified by Nenninger (1948) and should belong to the genus *Campanella*. Horst (2018) listed *O. tritora* in the synonyms of *C. umbellaria* but did not provide any comments on this misidentification. Due to the lack of important morphological data for *O. tritora*, further studies are needed to determine the correct taxonomy of this species.

Yu et al. (1995) reported a new *Campanella* species, *C. hanchuanensis*, from a freshwater pond in Hubei Province, China (Figure 6F). In the original description, evidence for the presence of a reticulate (vs. transverse) silverline system is ambiguous (Yu et al., 1995). However, in a subsequent report, Shen and Gu (2016) confirmed that the silverline system of *C. hanchuanensis* is reticulate. Although *C. hanchuanensis* closely resembles *C. umbellaria*, they can be separated as follows: (1) the stalk of *C. hanchuanensis* is curved, whereas that of *C. umbellaria* is straight and (2) the trochal band of *C. hanchuanensis* consists of six rows of kinetosomes with four compact middle rows and two separated peripheral rows, whereas that of *C. umbellaria* comprises two compact rows only (Stiller, 1941; Yu et al., 1995; Shen and Gu, 2016).

### Comparison of *Campanella sinica* n. sp. With Morphologically Similar Taxa

*Campanella sinica* n. sp. closely resembles *C. umbellaria* from which it can be separated as follows: (1) the zooid of *C. sinica* n. sp. is asymmetrical horn-shaped, while that of *C. umbellaria* is symmetrical and inverted bell-shaped; (2) the stalk of *C. sinica* n. sp. is smooth, whereas that of *C. umbellaria* has transverse annular bulges; and (3) the P3 of *C. sinica* n. sp. makes less than half a circuit within the infundibulum, whereas that of *C. umbellaria* makes 1–1.5 circuits. *Campanella sinica* n. sp. can be easily separated from *C. purneri* and *C. ovata* n. grad. & n. comb. by its asymmetrical horn-shaped (vs. elongated cylindrical) zooid, and convex, slightly elevated (vs. conspicuously elevated) peristomial disc. Compared with *C. hanchuanensis*, *C. sinica* n. sp. has a straight (vs. curved) stalk, asymmetrical horn-shaped zooids (vs. symmetrical inverted bell-shaped zooids), and the trochal band consists of several compact rows (vs. four compact middle rows and two separated peripheral rows). Based on these differences, *C. sinica* n. sp. can be easily distinguished from *C. hanchuanensis*.

### Key to the Identification of *Campanella* Species

The genus *Campanella* contains five valid species, i.e., *C. umbellaria*, *C. purneri*, *C. ovata* n. grad. & n. comb., *C. hanchuanensis*, and *C. sinica* n. sp. We here supply a key to their identification.

1 Zooid cylindrical                                    2

- Zooid inverted bell-shaped or asymmetric                        3

2 Stalk surface with several conspicuous transverse annular bulges        *C. purneri*

- Stalk surface smooth                               *C. ovata*

3 Zooid inverted bell-shaped                               4

- Zooid asymmetric                                *C. sinica*

4 Stalk straight                                *C. umbellaria*

- Stalk curved                               *C. hanchuanensis*

### Phylogenetic Analyses of the Genus *Campanella* and Other Sessilids

*Campanella* has been reported from freshwater habitats worldwide (Nenninger, 1948; Sládeček and Sládečková, 1974; Shen and Gu, 2016). Species of *Campanella* are characterized by their colonial lifestyle, dichotomously branched non-contractile stalk, everted multi-layered peristomial lip, and well-developed haplokinety and polykiney that make more than four circuits around the peristome before descending into the infundibulum. Traditionally, this genus has been assigned to the family Epistylididae because of the non-contractile stalk and the well-defined, everted peristomial lip (Corliss, 1979; Lynn, 2008). However, phylogenetic analyses based on molecular data have challenged this classification. Miao et al. (2004), for example, suggested that the genus *Campanella* should be independent of the family Epistylididae and probably represents a separate lineage based on phylogenetic analyses of SSU rDNA sequences. Nonetheless, this study was solely based on single gene analyses and did not provide morphological data to support this conclusion.

In the present study, *Campanella* nests within the basal clade of the sessilids and is distinctly separated from other epistylidids in all phylogenetic trees, which is consistent with the findings of Miao et al. (2004). Based on the present phylogenetic analyses, *Campanella* species show a close relationship with species of the family Operculariidae (Figures 1, 2A). However, operculariids are characterized by the absence of a peristomial lip, separating them from *Campanella* which has a conspicuous peristomial lip. Thus, the genus *Campanella* should not be assigned to the family Operculariidae. In two of the present phylogenetic trees, i.e., LSU rDNA and concatenated data, the closest relative of *C. sinica* n. sp. is the epistylidid *Pseudepistylis songi*, while in the SSU rDNA tree, these two species, along with another epistylidid, namely *Epistylis galea*, nest within the basal clade of the sessilids. Nevertheless, *Campanella* can be clearly separated from both *P. songi* and *E. galea* by its multi-layered (vs. single-layered) peristomial lip and the haplokinety and ploykinety which make more than four turns around the peristome (vs. less than two turns) before descending into the infundibulum (Foissner et al., 1992; Peng et al., 2007). Compared with all families within the order Sessilida, *Campanella* is morphologically distinguishable by its multi-layered peristomial lip and the unique oral ciliature. Thus, a combination of the morphological and molecular phylogenetic data suggests that the genus *Campanella* should belong to a separate family within the order Sessilida as proposed by Miao et al. (2004). However, the present and previous studies show that most families in the order Sessilida are non-monophyletic (Miao et al., 2004; Sun et al., 2012, 2013, 2016; Zhuang et al., 2018). Furthermore, *Epistylis galea*, *Pseudepistylis songi*, and *Rhabdostyla commensalis* are also independent of core epistylidids in the present phylogenetic trees. Therefore, the establishment of new family-level taxa for genera such as *Campanella* is premature and should await a re-evaluation of the family Epistylididae based on detailed morphological and accurate molecular data.

Although at least 800 nominal species of sessilids have been reported from various aquatic environments worldwide, the taxonomy and classification of this group are confusing (Foissner et al., 2010; Sun et al., 2016; Wang et al., 2017b; Zhang et al., 2019; Zhou et al., 2019a,b). The order Sessilida is subdivided into 14 families based on phenotypic characters, such as lifestyle modes (solitary or colonial), stalk structures (with or without spasmoneme), lorica (presence or absence), and living habits (sessile or free-swimming) (Lynn, 2008). However, the validity of several of these families has been challenged in recent years following the application of molecular phylogenetic analyses, mainly based on SSU rDNA sequence data (Miao et al., 2001, 2004; Utz and Eizirik, 2007; Utz et al., 2010; Zhan et al., 2013; Jiang et al., 2019). Miao et al. (2001) provided the first analyses of phylogenetic relationships within the subclass Peritrichia based on SSU rDNA sequences. Subsequent molecular phylogenetic studies have suggested that family or genus assignments of many taxa should be reevaluated (Clamp and Williams, 2006; Utz and Eizirik, 2007; Williams and Clamp, 2007). Members of the basal clade of the Sessilida assemblage (e.g., *Campanella umbellaria*, *Opercularia microdiscum*, and *Propyxidium* sp.) are thought likely to possess the plesiomorphic characters of sessilids (Sun et al., 2016). The results of the present study support the findings of previous studies that the family Epistylididae is polyphyletic and should be divided into several groups and/or various of its members should be re-assigned to other families (Sun et al., 2012, 2013; Zhou et al., 2019b; Lu et al., 2020). The clustering pattern in the phylogenetic trees suggests that the major epistylidid group (core *Epistylis*) evolved from species of the family Zoothamniidae, which indicates that these underwent loss of the stalk spasmoneme. It is noteworthy that in the tree based on the concatenated dataset, *E. chrysemydis* is nested within the Zoothamnidae thus supporting this hypothesis (Figure 2C). Zhuang et al. (2018) reported a population of *E. chrysemydis* with a hollow stalk containing a central bundle of fibers that is similar to the spasmoneme of *Zoothamnium*. Species of *Campanella* have a hollow stalk suggesting that stalks with this structure may also represent an ancestral trait of the sessilids. Furthermore, based on their gene sequence similarities, *Campanella* spp. (represented by SSU rDNA sequence KU343248 and ITS1-5.8S rDNA-ITS2 sequence GU586188) and *C. sinica* n. sp. are probably conspecific, although it is not possible to verify this due to the lack of vouchered specimens or morphological data for the former taxa.

## Data Availability Statement

The datasets presented in this study can be found in online repositories. The names of the repository/repositories and accession number(s) can be found at: https://www.ncbi.nlm.nih.gov/genbank/ (MW969624, MW969627, and MW969625).

## Author Contributions

ZW, LL, and CW conceived and designed the paper. ZW and TW carried out the live observations, protargol staining, and phylogenetic analyses. ZW, TW, BL, AW, YC, XZ, SA-F, LL, and CW wrote the paper. All authors contributed to the article and approved the submitted version.

## Conflict of Interest

The authors declare that the research was conducted in the absence of any commercial or financial relationships that could be construed as a potential conflict of interest.

## Publisher’s Note

All claims expressed in this article are solely those of the authors and do not necessarily represent those of their affiliated organizations, or those of the publisher, the editors and the reviewers. Any product that may be evaluated in this article, or claim that may be made by its manufacturer, is not guaranteed or endorsed by the publisher.

## Footnotes

^1^ http://tree.bio.ed.ac.uk/software/figtree/
